# Sucrose Matters. The Need to Make Groups Truly Comparable When Assessing Changes Associated with Insulin Sensitivity. Comment on “Consumption of Cooked Black Beans Stimulates a Cluster of Some Clostridia Class Bacteria Decreasing Inflammatory Response and Improving Insulin Sensitivity.” *Nutrients* 2020, *12*(4), 1182

**DOI:** 10.3390/nu12072091

**Published:** 2020-07-16

**Authors:** Jorge Cervantes

**Affiliations:** Texas Tech University Health Sciences Center, El Paso, TX 79905, USA; jorge.cervantes@ttuhsc.edu

The recent paper by Sanchez-Tapia et al. [1] offers interesting results that should be taken with caution due to problems in the study design. The study compares groups of rats fed with Casein (C) or cooked Black Beans (BB), with and without a high-fat (HF) diet (i.e., 17g of lard). The main problem with the methodology lies in the composition of the diets, as they differ not only in the addition of lard but also in the amount of casein (present only in the C and C+HF group); the amount of cornstarch (41 vs. 23.97 in the C and C+HF groups, respectively); and, most notably, the amount of sucrose provided to the animals. Even though their composition of lipids, proteins, and carbohydrates are similar, the high amount of lard does have an effect on hypothalamic insulin signaling [2]. Authors show in their Table that C and C+HF groups received 10 and 7.8 g of sucrose in the diet, while BB and BB+HF received 1.3 g and 0 g of sucrose, respectively. Despite the total carbohydrate content being similar, the difference in sucrose intake is of outmost importance as the effect of sucrose in insulin response has been long known [3,4]. This brings problems with the Results, as this sucrose intake difference could certainly explain the findings of rats on C and C+HF gaining significantly more weight and higher glucose levels than the BB and BB+HF groups, shown in the study.

The crucial differences in sucrose between the compared groups make the conclusions made in this paper rather unreliable, as insulin [5], leptin [6], and intestinal microbiota changes [7] can all be a consequence of higher glucose.

Another problem with the data shown in the paper is the claim made by the authors of the highest abundance of *Ruminococcus, Coprococcus,* and *Prevotella,* in the BB group, compared to other groups at the Genus level, despite their LDA data (at the species level) not showing *Prevotella* at all. The presence of this bacterium is important given the fact that increased abundance of *Prevotella* is associated with improved glucose metabolism [8], and in this case, an artifact due to the difference in sucrose intake and subsequent glucose levels among the groups.

The authors make the assertion that LPS is more associated with gut microbiota, despite their own data showing more Firmicutes (Gram-positive) than Bacteroidetes (Gram-negative) across all groups. Differential abundance of certain Gram-negative or Gram-positive across the groups does not necessarily explain an increase in serum LPS, as this requires invasiveness of such Gram negatives to make such a claim valid. Other assertions in the paper, such as high-fat diet increasing the concentration of LPS associated with high intestinal permeability, are also questionable. Although a high-fat diet can enhance intestinal permeability [9], this may not necessarily be associated with an increased concentration of LPS. Had an effect of high fat on LPS occurred, higher LPS values in the BB + HF than in BB alone would have been observed. This, however, was not the case (Figure 3G).

The paper’s discussion highlights the many beneficial aspects of SCFA, which is rather odd, as the BB group did not show the highest total SCFA, compared to C+HF or C groups. It also portrays that the improvement in glucose levels is due to the consumption of BB, which may not be true for the reasons already explained. Such directionality of presenting their findings, without supporting data, could lead to a misleading impression in the readers of mechanisms that a cooked BB diet simply does not elicit.
